# Shedding light on the Chimaeridae taxonomy: the complete mitochondrial genome of the cartilaginous fish *Hydrolagus mirabilis* (Collett, 1904) (Holocephali: Chimaeridae)

**DOI:** 10.1080/23802359.2020.1870887

**Published:** 2021-02-09

**Authors:** André Gomes-dos-Santos, Nair Vilas-Arrondo, André M. Machado, Ana Veríssimo, Montse Pérez, Francisco Baldó, L. Filipe C. Castro, Elsa Froufe

**Affiliations:** a CIIMAR/CIMAR – Interdisciplinary Centre of Marine and Environmental Research, Terminal de Cruzeiros de Leixões, Av. General Norton De Matos s/n, University of Porto, Matosinhos, Portugal; b Department of Biology, Faculty of Sciences, University of Porto, Porto, Portugal; c Instituto Español de Oceanografía, Centro Oceanográfico de Vigo, Vigo, Spain; d Faculty of Biology, UVIGO, PhD Program “Marine Science, Technology and Management” (Do *MAR), University of Vigo, Vigo, Spain; e CIBIO – Research Centre in Biodiversity and Genetic Resources, Vairão, Portugal; f Instituto Español de Oceanografía, Centro Oceanográfico de Cádiz, Puerto Pesquero, Muelle de Levante s/n, Cádiz, Spain

**Keywords:** Chondrichthyes, Holocephali, chimaera, mitogenome, phylogenetics

## Abstract

Cartilaginous fish are fascinating taxa, present in the folklore and art of many different cultures. Moreover, they display several unique anatomical, physiological, molecular, and behavioral characteristics making them extremely interesting from a biological perspective. Nevertheless, some crucial knowledge gaps remain, including phylogenetic relationships among extant species. Here, we produced the complete mitogenome sequence of the large-eyed rabbitfish, *Hydrolagus mirabilis* (Chimaeriformes). The complete mitogenome is 19,435 bp long and shows the same overall content, i.e. 13 protein-coding genes, 22 transfer RNA, and two ribosomal RNA genes, as all other examined Chondrichthyan mitogenomes. Phylogenetic reconstructions including 12 Holocephalan and three outgroup Elasmobranch mitogenomes place the *H. mirabilis* within the family Chimaeridae but revealed paraphyletic *Hydrolagus* and *Chimaera*, in line with a previous study, highlighting the importance for collecting additional molecular data to improve phylogenetic reconstruction in this group of vertebrates.

Cartilaginous fish (Chondrichthyes), i.e. sharks, rays, and chimaeras, are extremely interesting from a biological perspective as they represent one of the oldest and most ecologically diverse groups of jawed vertebrates (Boisvert et al. 2019). Importantly, they show several unique anatomical, physiological, molecular, and behavioral characteristics (see Lopes-Marques et al. 2015; Boisvert et al. 2019). The Holocephali (chimaeras and ratfish), one of the two subclasses of the Chondrichthyes, includes the Family Chimaeridae which comprises the genus *Hydrolagus* with more than 20 described species (James et al. 2009; Freitas et al. 2011; Catarino et al. 2020). Yet, the paucity of molecular data from this group of gnathostomes impairs a clear evolutionary outline of the relationships between extant lineages. Mitogenomes have been a powerful tool used to elucidate phylogenetic relationships, both at deep and at shallow evolutionary nodes (e.g. Inoue et al. 2010; Gomes-dos-Santos et al. 2020).

*Hydrolagus mirabilis* (Collett, 1904) (Holocephali: Chimaeridae), commonly known as the large-eyed rabbitfish, is widely distributed in the Atlantic Ocean, and in the Mediterranean Sea, generally occupying depths over 800 m. At the moment, *H. mirabilis* is not a commercial fishing target, being caught accidentally in deep-sea trawl, longline, and gillnet fisheries. Currently, the species is listed as a Least Concern by the IUCN (Finucci 2020). Nevertheless, once regarded only as by-catch species, Chondrichthyans are increasingly represented in the fisheries of most countries and are among the most imperilled marine organisms, with up to a quarter of the species facing an increased risk of extinction (Dulvy et al. 2014; Davidson et al. 2016).

A male *H. mirabilis* specimen was captured in the Porcupine Bank (NE Atlantic) on 11 September 2019 at 51.0316°N, 14.4061°W and 751 m depth during the Spanish Bottom Trawl Survey PORCUPINE 2019. The initial morphological identification was performed on board. Genomic DNA was extracted and used for whole-genome sequencing of 150 bp paired-end (PE) reads on a Hiseq X Ten.

To obtain the complete mitogenome, an assembly was produced with SPAdes (v3.12.0) (Bankevich et al. 2012) using 10% of the total PE reads. Afterwards, the complete mitogenome was retrieved by blast search (Altschul et al. 1990) against all Teleostei mitogenomes available on GenBank. Annotation was performed using MitoZ (v.2.3) (Meng et al. 2019).

All Holocephali mitogenomes publicly available (12 sequences), as well as three outgroup Elasmobranchii mitogenomes (i.e. *Squalus brevirostris*, *Carcharhinus amblyrhynchos*, and *Raja radiata*) were retrieved from GenBank (12-09-2020) and their 13 protein-coding genes (PCGs) aligned and concatenated using GUIDANCE (v.1.5) (Penn et al. 2010) and FASconCAT-G (https://github.com/PatrickKueck/FASconCAT-G), respectively (final length: 11,431 bp). The best partition-scheme for each gene and maximum-likelihood (ML) phylogeny were obtained using IQ-TREE (v.1.6.12) (Nguyen et al. 2015; Kalyaanamoorthy et al. 2017).

The complete mitogenome of *H. mirabilis* was deposited in GenBank (MW029477). The mitogenome length is 19,435 bp, which is within the expected length for holocephalans (16,758–24,889 bp), and the gene composition is in agreement with that of vertebrate mtDNA: 13 PCGs, 22 transfer RNA, and two ribosomal RNA genes. One PCG (NAD6) and eight tRNAs are encoded on the light strand. Furthermore, a Chimaeriformes-specific long noncoding insertion (2215 bp) between tRNAThr and tRNAPro genes is also present (Inoue et al. 2010).

The phylogenetic tree (Figure 1), recovered the two major Chondrichthyan subclasses as reciprocally monophyletic, i.e. Holocephali and Elasmobranchii.

**Figure 1.**
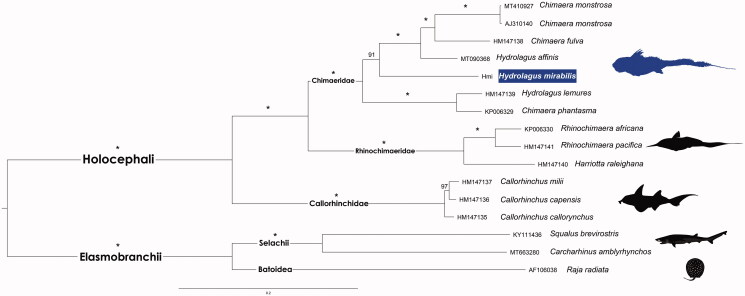
Maximum likelihood phylogenetic tree based on 13 concatenated protein-coding genes of 12 Holocephali and three outgroups Elasmobranchii mitogenomes. GenBank accession numbers are shown ahead of species names. The * above the branches indicates both posterior probabilities and bootstrap support values above 99%.

As previously described, the Callorhinchidae, Rhinochimaeridae, and Chimaeridae families were retrieved as three well-supported clades within Holocephali (Inoue et al. 2010). The paraphyletic status of *Hydrolagus* and *Chimaera* was also recovered, as recently reported by Gomes-dos-Santos et al. (2020). The newly sequenced *H. mirabilis* mitogenome ranges in the percentage of sequence divergence from 12.8% (unc *p*-distance) from *H. affinis* to 15.4% from *H. lemures*. The prevalence of this paraphyly in the Chimaeridae, reinforce the scenario of a possible misidentification of one of the specimens, most likely *Chimaera phantasma*. Yet, the authors are engaged at raising the few existing molecular data from this group of gnathostomes to clarify the evolutionary relationships between extant lineages.

## Data Availability

The data produced in this study are available in GenBank of NCBI at https://www.ncbi.nlm.nih.gov, reference number MW029477 or from the corresponding authors, L. Filipe C. Castro and Elsa Froufe.
